# Decreased wheel-running activity in hamsters post myocardial infarction

**DOI:** 10.1186/1479-5876-4-51

**Published:** 2006-12-02

**Authors:** Stefan Schäfer, Wolfgang Linz, Katja Hürland

**Affiliations:** 1 Cardiovascular Research Institute, Bayer Healthcare AG, Wuppertal, Germany; 2 Therapeutic Department Cardiovascular Diseases; Aventis Pharma Deutschland GmbH, Frankfurt am Main, Germany; 3 Institut für Veterinärphysiologie, Justus-Liebig-Universität, Giessen, Germany

## Abstract

Reduced exercise capacity is a key symptom and an independent determinant of mortality in patients with heart failure. We analyzed the running activity of hamsters with cardiac dysfunction after myocardial infarction.

In 39 male Syrian hamsters aged 10 to 12 weeks, a myocardial infarction (MI) was produced by permanent ligation of the left coronary artery. Spontaneous running activity in a wheel was monitored daily. After four weeks, left ventricular (LV) hemodynamics (catheter tip manometry) were measured at baseline and during inotropic stimulation (isoprenaline 0.03, 0.1 and 0.3 μg/kg/min i.v.). LV infarct size was quantified using planimetry.

Four weeks post MI, daily running distance was reduced stepwise in animals with small (4–15 % of LV: 9.8 ± 3.4 km/d) and large (> 15 % of LV: 7.5 ± 3.5 km/d) MI, compared to sham-operated hamsters (11.5 ± 1.5 km/d). Similar reductions were observed in maximum speed and distance of longest running period. MI size influenced daily running distance, maximum speed, and longest running period (linear correlations, all p < 0.05). MI size also impaired LV systolic and diastolic function under isoprenaline stimulation.

The results suggest that myocardial infarction reduces running capacity and isoprenaline stimulated LV function in hamsters, mimicking impaired exercise performance in patients with heart failure. Analysis of running activity in hamsters with myocardial infarction offers a unique opportunity for non-invasive and serial functional assessment of heart failure in the experimental setting.

## Background

Heart failure (HF) is a clinical syndrome in which the heart loses its ability to pump blood efficiently [1]. A key clinical symptom is shortness of breath upon physical exertion, depending on the severity of the disease. Consequently, the New York Heart Association (NYHA) classification, which is based on a semi-quantitative assessment of exercise capacity, has been established as a pragmatical, functional parameter to quantify the degree of HF in any individual patient. Interestingly, the NYHA classification remains an independent predictor of mortality, despite the increasing availability of more sophisticated functional and morphological indices for the assessment of HF [1].

In animals, myocardial dysfunction can be induced by a large number of different methods reflecting the different etiologies of HF in the clinic. Among those, the post myocardial infarction (MI) model in rodents, originally introduced by Pfeffer et al. [2], has been accepted to reflect the patho-physiology of myocardial remodeling during the evolution of HF very accurately. However, functional parameters are difficult to obtain in these animals. Whereas invasive measurement of LV function requires deep anesthesia and is limited to a single end point examination, the use of non-invasive imaging by echocardiography has been reported to be rather insensitive.

In the present study, we quantified daily wheel-running performance in hamsters with and without myocardial infarction. Unlike other rodents, the hamster has a genetic drive to run long distances spontaneously, which avoids variability in exercise performance due to learning and training effects. Our results suggest that, similar to the clinical situation, the degree of cardiac dysfunction in hamsters can be quantitatively assessed using activity in the running wheel. The hamster post MI model therefore offers a unique experimental setup to study the functional aspects of HF serially and non-invasively.

## Experimental Procedures

The current study was performed as part of K.H.'s DVM thesis at Aventis Pharma, Frankfurt am Main, Germany. The animal experiments were performed in accordance with Aventis Laboratory Animal Science and Welfare guidelines and the German law for the protection of animals.

### Animals and laboratory conditions

Male Syrian hamsters were purchased from Harlan-Winkelmann GmbH (Borchen, Germany) and arrived in the animal care facilities at the age of 8–10 weeks. The animals were housed individually in cages which were permanently equipped with a running wheel (diameter 17 cm). A 12:12-h light-dark cycle was kept throughout the experimental period. Ambient temperature and humidity were kept constant at standard conditions. Food pellets and water were provided ad libitum.

### Wheel-running activity

The wheel-running activity was monitored continuously in 5 min segments by electromagnetic induction using a specialised data acquisition system (V1.48, Ingenieurbüro Jäckel, Hanau, Germany), connected to a personal computer. Microsoft Excel was used for data processing. After an acclimatisation period of at least ten days, baseline data were taken as the mean of 3 days. After surgical induction of the myocardial infarction (MI, vide infra), the hamsters were taken back into their cages and wheel-running activity was followed up for four weeks. Hamsters were considered active when more than 20 rotations per 5 min were recorded. Using the raw wheel rotation data, the following parameters were calculated: total daily running distance (in km), maximum running speed (in km/h) and longest running period (in km). In order to account for inter-day variability, daily data were averaged for statistical analysis over three days at baseline and weekly post MI. In addition, in order to assess the fragmentation of the running behavior, daily activity records (actograms) were produced from the day before the terminal experiments, i.e., approximately 4 weeks post MI.

### Surgical induction of MI

The animals were anesthetized with isoflurane (3 % v:v) plus fentanyl (33 μg/kg i.p., Essex Pharma, Munich, Germany) and artificially ventilated (60 times 1.2 mL room air per minute). After opening the chest through the third intercostal space, a myocardial infarction was produced by permanent ligation of the left coronary artery, approximately 5 mm distal to its origin from the aorta. Animals randomly assigned to sham underwent the identical experimental procedure except that no ligation of the coronary artery was produced. Recovery of the animals from the operation was supported by local anesthesia at the sites of incision (xylocain 1%, Astra Zeneca, Wedel, Germany) and buprenorphin (0.1 mg/kg i.p., Essex Pharma, Munich, Germany) at the end of the procedure. In addition, the animals were supplemented with furosemide, (initial dose 6 mg/kg, 4 mg/kg/day, Aventis Pharma), metamizol (400 mg/kg/day, Aventis Pharma), and enrofloxazine (2.5 mg/kg/day, Bayer, Leverkusen, Germany) via the drinking water for the first 5 post-operative days.

### Echocardiography

At two weeks post MI, a transthoracic echocardiography (M-mode) was performed on all hamsters (HDI3000, ATL, Solingen, Germany) under light anesthesia (1.5 vol% isofluran). Left ventricular dimensions as well as end diastolic septal and posterior wall thicknesses were measured in a parasternal short axis view at the level of the papillary muscles, according to the leading-edge convention of the American Society of Echocardiography. LV fractional shortening (FS) was calculated as: FS = (EDD – ESD)/EDD where EDD and ESD represent LV end-diastolic and end-systolic dimension, respectively.

### Terminal experiments

At four weeks post MI, the hamsters were again anesthetized with isoflurane (1.5 % v:v) plus fentanyl (33 μg/kg i.p.) and artificially ventilated. A catheter tip-manometer (Millar Instruments, Houston, Tx., USA) was introduced into the left ventricle via the carotid artery. Heart rate, left ventricular pressure, its derivative dP/dt and the relaxation constant τ [3] were then measured at baseline and during steady state i.v. infusion of isoprenaline (0.03, 0.1, and 0.3 μg/kg/min, Sigma, Steinheim, Germany). After the hemodynamic measurements, the animals were killed by quick excision of the hearts. The LV (excluding the septum) was isolated from the heart and the infarct size (as percent of LV) quantified planimetrically.

### Statistics

In a pilot study, we had found that mortality was extremely high in hamsters with MI sizes > 30 % of the LV (details not shown). We therefore arbitrarily defined the middle value between 0 and 30 % (i.e., 15 %) as the threshold MI size to distinguish between the small and large infarct size groups. Because we expected a large variability in running performance after the first operation, it was predefined to perform statistical testing at baseline and at the end of the experiments (i.e., four weeks post MI). Differences between groups were tested for significance by two-way ANOVA, followed by Bonferroni posttests. Correlations between MI size (as independent variable) and running parameters or hemodynamic indices (as dependent variables) were calculated using a linear model. Data are reported as mean +/- SD, unless otherwise stated. A p value of less than five per cent was considered significant.

## Results

### Running activity

**Figure 1:**
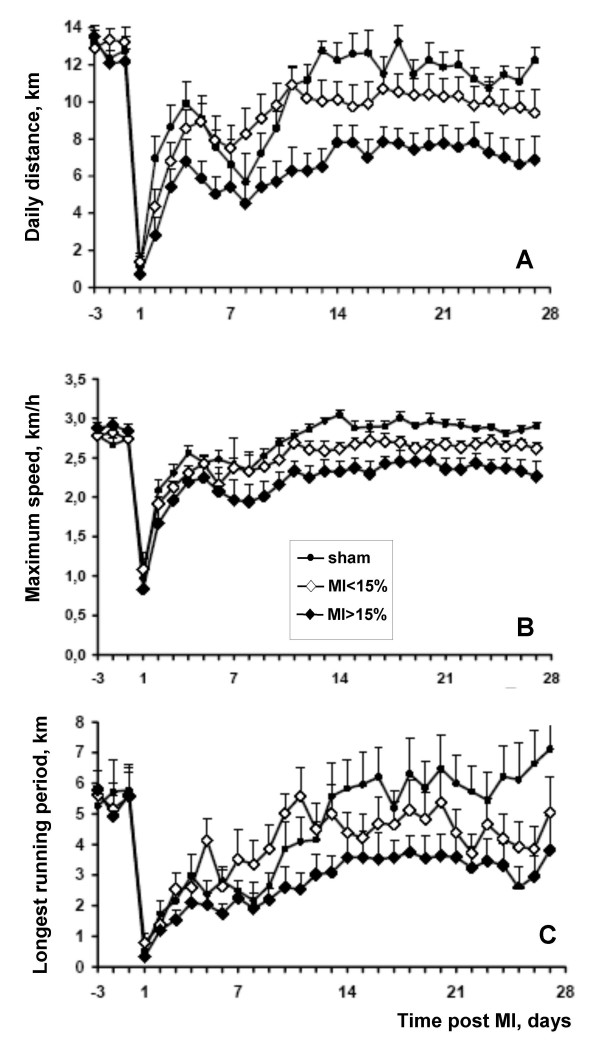
Daily running distance (panel A), maximum speed (panel B), and longest running period (panel C) over time in sham-operated hamsters (dots) and in hamsters with small (< 15 % of LV, open diamonds) and large MIs (> 15%, filled diamonds). Data are mean ± sem.

**Figure 2:**
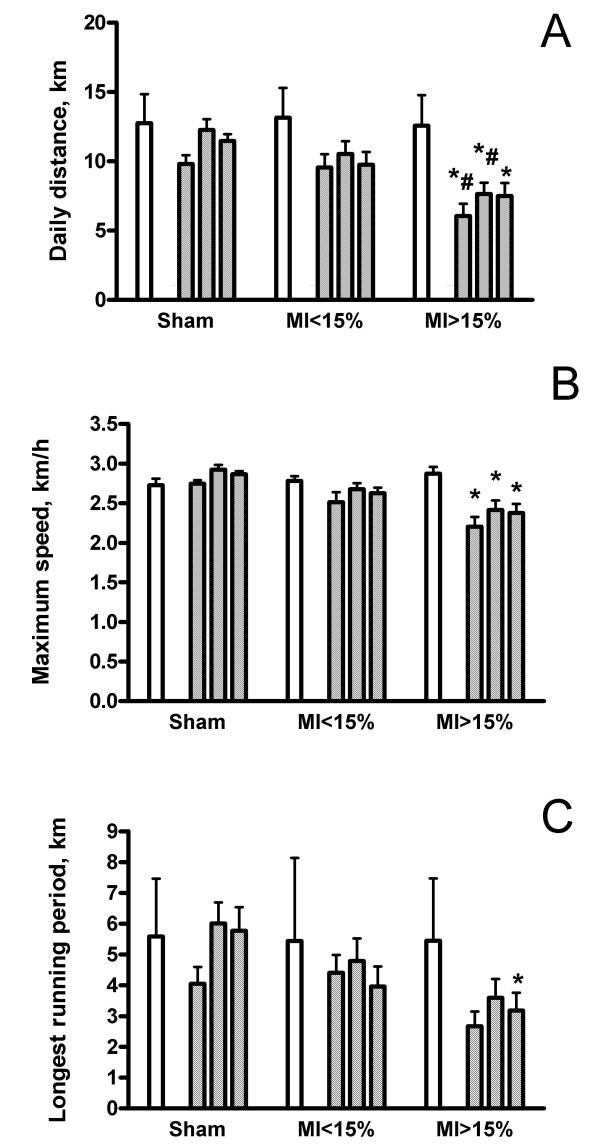
Daily running distance (panel A), maximum speed (panel B), and longest running period (panel C) at baseline (open bars), and weekly during weeks 2, 3, and 4 after MI (hatched bars). Data are mean ± sd. Data are grouped into sham-operated hamsters (n = 10) and hamsters with small (< 15 % of LV, n = 15) and large MIs (> 15%, n = 14).

The time course of daily distance, maximum speed, and longest running period at baseline and during the post MI period are shown for the three groups in figure 1. In animals with large MI, all three parameters were markedly reduced compared to sham animals. In the small MI group, the reduction in running performance was less pronounced. Figure 2 illustrates the weekly exercise performance at baseline and during weeks 2, 3 and 4 post MI. The data for the first postoperative week are not shown, because of the large variation during this recovery period. In the large MI group, daily distance, maximum speed, and longest running period were significantly reduced at all times (2 to 4 weeks) post MI.

**Figure 3:**
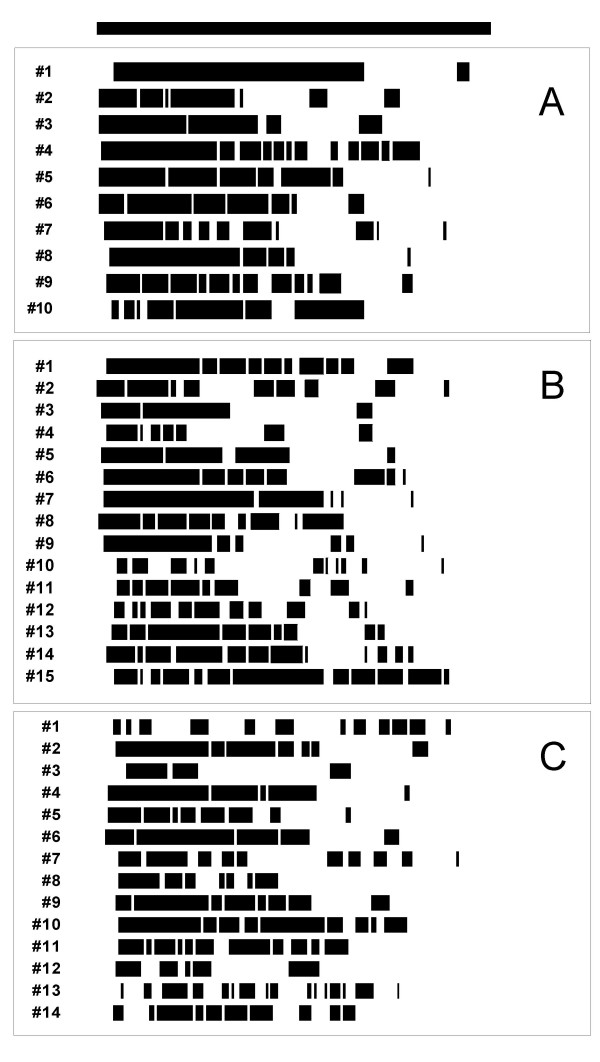
Wheel-running actograms at four weeks post MI from individual hamsters in the sham (panel A), small MI (panel B), and large MI (panel C) groups. The dark bar on top indicates the dark period (12 hours). Black bars in individual lines represent running activity, white spaces pauses. Animals with MI show a more fragmented activity pattern than sham-operated animals.

Another aspect of daily activity is illustrated by the actograms from all individual animals at four weeks post MI (figure 3). There was a more fragmented activity pattern in animals with MI, compared to sham-operated animals, with no major difference between the small and large MI groups. This impression is reflected by a moderate (albeit statistically not significant) tendency towards a smaller number of activity bouts in the sham (7.3 ± 3.3 bouts/d), compared to the small MI (8.3 ± 2.9 bouts/d) or large MI (8.3 ± 3.4 bouts/d) groups.

### Echocardiography

The left ventricular systolic, but not diastolic diameter was increased in hamsters with large MIs. The end diastolic wall thicknesses were reduced in the groups with small or large MIs, compared to the sham group. Fractional shortening was significantly reduced in the animals with MI, compared to sham (table 1).

**Table 1:** Echocardiographic parameters

		**Sham**	**Small MI**	**Large MI**
		
LVDed	mm	5.3 ± 0.3	5.3 ± 0.8	5.7 ± 0.8
LVDsys	mm	3.9 ± 0.6	4.4 ± 1.2	5.1 ± 0.5 *#
Septum thickness	mm	0.8 ± 0.1	0.6 ± 0.1 *	0.6 ± 0.1 *
Posterior wall thickness	mm	0.9 ± 0.1	0.6 ± 0.1 *	0.6 ± 0.1 *
Fractional shortening	%	28.7 ± 2.4	16.0 ± 2.3 *	10.4 ± 2.2 *

Values are mean ± sd, n = 10 (Sham), n = 14 (MI groups). LVDed, left ventricular end diastolic diameter; LVDsys, left ventricular end systolic diameter; *, p < 0.05 vs. Sham; #, p < 0.05 vs. MI < 15%.

### Terminal experiments

LV hemodynamics during the terminal experiments are shown in table 2. Stepwise infusion of isoprenaline tended to increase LVdP/dtmax, an index of contractility, in the sham and the small MI group, but not in the large MI group. Isoprenaline infusion amplified the hemodynamic differences between the groups. For example, LVPmax was significantly reduced in the large MI group compared to sham during isoprenaline infusion (0.3 μg/kg/min), but not at baseline. Similarly, the differences in dP/dtmax between the small and large MI groups became significant during isoprenaline infusion (table 2).

**Table 2:** Invasive Hemodynamics

		**Isoprenaline, μg/kg/min**		
		
	**Baseline**	**0.03**	**0.1**	**0.3**
	
	**Heart rate, bpm**			
	
Sham	441 ± 70	455 ± 79	451 ± 77	431 ± 87
MI < 15%	389 ± 45	423 ± 38	427 ± 50	410 ± 54
MI > 15%	376 ± 53 *	390 ± 46	387 ± 49	369 ± 67
	**LVPmax, mmHg**			
	
Sham	117.1 ± 12.1	113.4 ± 13.4	118.0 ± 11.0	118.8 ± 12.1
MI < 15%	108.8 ± 15.1	107.6 ± 16.2	108.2 ± 18.0	110.7 ± 18.1
MI > 15%	102.0 ± 15.4	102.2 ± 11.4	103.1 ± 13.4	99.1 ± 9.3 *
	**LVEDP, mmHg**			
	
Sham	5.0 ± 3.2	4.6 ± 3.8	5.1 ± 3.4	8.6 ± 4.0
MI < 15%	9.2 ± 6.9	9.1 ± 7.3	10.4 ± 8.4	13.3 ± 8.5
MI > 15%	14.0 ± 6.2 *	15.5 ± 6.9 *	16.9 ± 7.9 *	18.4 ± 8.8 *
	**dP/dtmax, mmHg/s**			
	
Sham	7795 ± 2150	7992 ± 2110	8692 ± 2283	8220 ± 1560
MI < 15%	6365 ± 1458	6807 ± 1802	7319 ± 2184	7554 ± 2406
MI > 15%	5010 ± 1171 *	5137 ± 1626 *	5234 ± 1466 *#	5091 ± 1490 *#
	**dP/dtmin, mmHg/s**			
	
Sham	5954 ± 1863	5870 ± 1506	6153 ± 1498	6002 ± 1676
MI < 15%	4793 ± 1530	4878 ± 1360	5028 ± 1642	4887 ± 1187
MI > 15%	3851 ± 1041 *	3876 ± 1130 *	3881 ± 1012 *	3667 ± 848 *
	**τ, ms**			
	
Sham	14.1 ± 8.0	11.9 ± 6.0	12.6 ± 6.4	12.2 ± 5.1
MI < 15%	15.7 ± 7.4	13.7 ± 7.4	12.1 ± 5.4	11.3 ± 3.8
MI > 15%	17.9 ± 7.2	18.4 ± 8.4	17.5 ± 7.5	15.7 ± 4.9

Values are mean ± sd, n = 8 (sham), n = 14 (MI groups). *, p < 0.05 vs. Sham; #, p < 0.05 vs. MI < 15%.

### Impact of MI size on exercise capacity and hemodynamics

**Figure 4:**
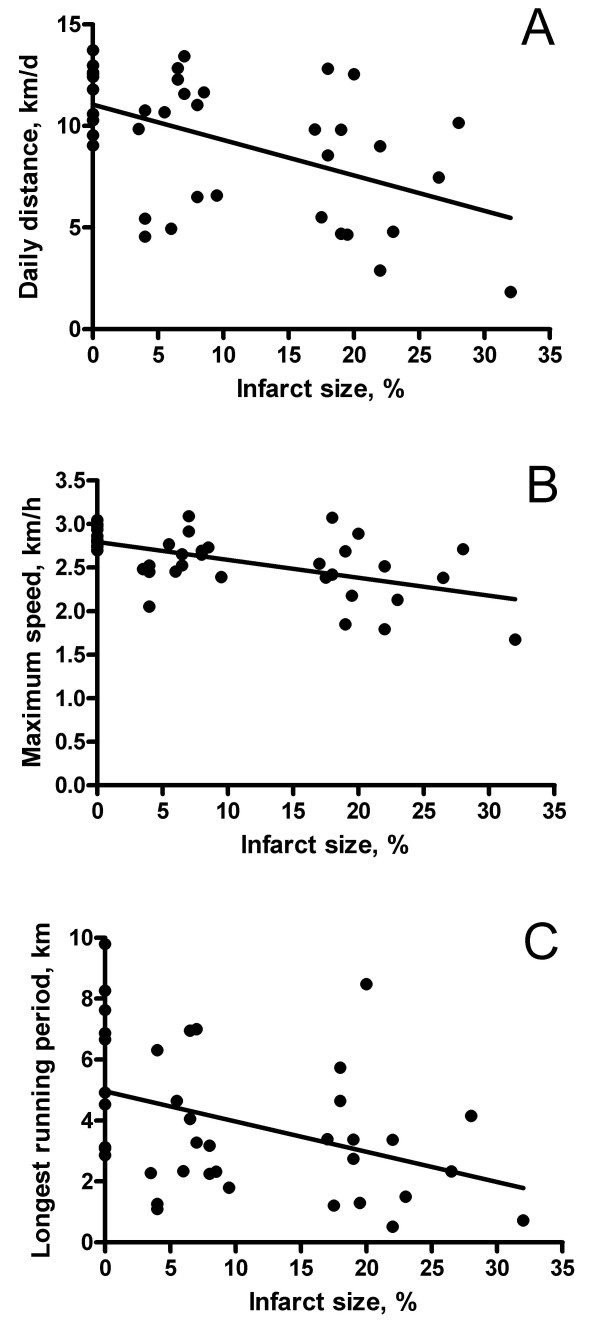
Scatterplots of infarct size vs. exercise parameters in all hamsters. There were linear correlations of infarct size vs. daily distance (panel A, r = 0.50), maximum speed (panel B, r = 0.56), and longest running period (panel C, r = 0.39); all p < 0.05.

**Figure 5:**
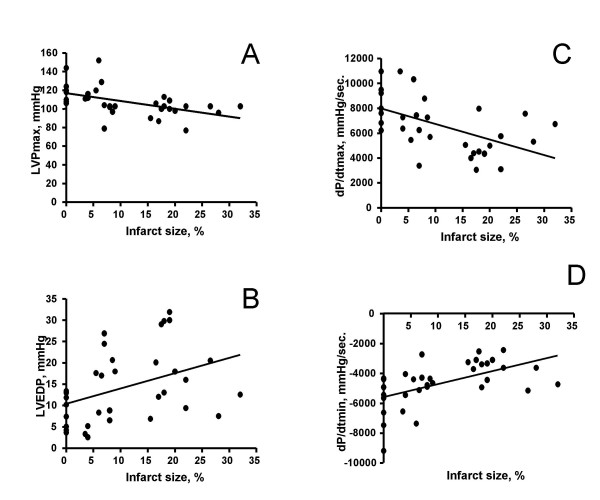
Scatterplots of infarct size vs. hemodynamic parameters during inotropic stimulation (isoprenaline 0.3 μg/kg/min i.v.) in all hamsters. There were linear correlations of infarct size vs. LVPmax (panel A, r = 0.51), LVEDP (panel B, r = 0.39), LVdP/dtmax (panel C, r = 0.53), and LVdP/dtmin (panel D, r = 0.55); all p < 0.05.

MI size was a significant predictor of daily running activity, as demonstrated in figure 4. There were also siginificant linear correlations between MI size and indicators of LV systolic (LVPmax, dP/dtmax), as well as diastolic (LVEDP, dP/dtmin) function. Figure 5 shows the respective scatterplots during isoprenaline stimulation (0.3 μg/kg/min). At baseline, the correlations between MI size and the hemodynamic parameters were also statistically significant (p < 0.05), but less strong (details not shown).

## Discussion

The present study demonstrates that activity in the running wheel is reduced in hamsters with chronic myocardial infarction, corresponding to impaired exercise performance in patients with HF. Therefore, analysis of running activity in hamsters with cardiac dysfunction offers a unique opportunity for non-invasive and serial functional assessment of experimental heart failure.

Whereas in the clinical setting the degree of exertional dyspnea (assessed using the NYHA classification or quantified with spiroergometry) has been established as both a marker of severity and a predictor of mortality in heart failure, the effect of myocardial infarction on spontaneous exercising has not yet been investigated in animals. In the present study, we used the post MI model to induce cardiac dysfunction because MI is the most frequent single cause for HF in patients [4]. Originally described in rats [2], MI induced heart failure has subsequently been demonstrated in a number of other laboratory species. Coronary ligation has been described to result in myocardial infarction in hamsters, too [5] but there have been no reports on routine physical activity in hamsters with chronic myocardial infarction so far. In the present study, myocardial infarction was confirmed at necropsy, and the presence of cardiac dysfunction was verified using a number of echocardiographic and invasive hemodynamic parameters (cf. tables 1 and 2).

Compared to an amazing average daily running distance of approximately 13 km at baseline, post MI heart failure resulted in reduction of up to 50 per cent, depending on the MI size. Similarly, both peak running speed and endurance were reduced by approximately 25 and 50 per cent, respectively. We estimate that this reduction can be compared to a clinical severity of NYHA class II-III. Moreover, in our experimental series, several animals were dyspneic and not able to run at all after the MI operation (clinically resembling NYHA IV) and did not survive the four weeks follow-up. These hamsters were noted to have very large MIs upon necropsy (> 30 per cent) and were therefore not included in the statistics.

Penev et al. have previously performed an analysis of age-related fragmentation of running activity in hamsters [6]. They discovered that running activity is markedly reduced in old (age 18–19 months), compared to young (age 8–10 weeks) animals. In addition, the running activity was much more fragmented (i.e., more but shorter periods of continuous running) in the old vs. the young group. The interesting question ensues whether the decrease in activity was due to heart failure or other factors, such as age related disturbance of circadian organization. Unfortunately, no invasive hemodynamic measurements to assess cardiac function have yet been reported in old hamsters. It is interesting to note, however, that while fragmentation and reduction in daily running distance were similar in the aging and the post MI hamsters, peak running speed is reduced in post MI but not in aging hamsters. Exercise testing has been used to quantify the functional capacity of patients with heart failure, and a reduction in peak exercise capacity or peak oxygen uptake reflects a worsening prognosis [1]. It seems therefore plausible to assume that in contrast to the aging hamster, heart failure is the major determinant for the alterations in physical activity after a myocardial infarction. Interestingly, an upregulation of the vasopressin gene in the suprachiasmatic nucleus is related decreased locomotor activity of tau mutant hamsters (which are characterized by a shorter circadian rhythm) [7]. Plasma vasopressin concentrations are known to be elevated in heart failure [8], and it will be interesting to test whether plasma or brain vasopressin levels determine exercise activity in hamsters with heart failure.

The correlations between infarct size and either running activity or hemodynamic parameters were found to be relatively weak in the present study, and were not observed within the groups. This observation is not surprising, though, given that in patients, infarct size and degree of cardiac dysfunction do not correlate well with heart failure symptoms. Obviously, the extent of heart failure as a clinical syndrome is determined by additional factors, such as the level of neuro-endocrine activation, myocardial remodeling, or the duration of the disease.

While myocardial infarction and aging undoubtedly have an influence on wheel running activity in hamsters, the level of physical activity may also have an impact on cardiovascular function and prognosis. In humans, a sedentary life style is an accepted cardiovascular risk factor, and physical activity can improve outcome in patients with heart failure [9]. Similarly, chronic desynchronization of the dark-light cycle, which may quantitatively impair physical activity, has been shown to increase mortality in cardiomyopathic hamsters [10]. It remains to be investigated, however, whether an increase in wheel running activity can improve cardiovascular function in hamsters with heart failure.

In contrast to hamsters, voluntary wheel running in rats is characterised by a large variability, both over time and inter-individually [11]. Moreover, it is associated with a marked decrease in body fat tissue, indicating a training effect over time. Similarly, voluntary wheel running has been reported to decrease body weight in mice [12] and to enhance the anorectic effects of a cannabinoid recepor antagonist [13]. By comparison, access to the running wheel increases body and organ masses in the hamster without a change in body composition and improves the general health status of golden hamsters under laboratory conditions [14]. Hamsters are highly motivated to use running wheels. Wheel running starts almost immediately after onset of the dark period (cf. Figure 2), it is independent of the environment in the cage (enriched or monotonous) [15], and it steadily decreases with advancing age [14,6]. In addition, our own (unsystematic) observations indicate that removing the running wheel from the cages results in stereotypical movements (such as repetitive jumping at the wall of the cage), which is a typical consequence of confining animals with a wide-ranging natural lifestyle [16]. Thus, although it has been proposed that wheel running may be an artifact of the captive environment [17], it nevertheless contributes to the well being of the animals. In conclusion, therefore, the hamster seems an ideal animal model to assess the consequences on exercise capacity of a wide variety of experimental interventions serially and non-invasively.

## Competing interests

The author(s) declare that they have no competing interests.
